# Characteristics of patients treated by the Danish Helicopter Emergency Medical Service from 2014-2018: a nationwide population-based study

**DOI:** 10.1186/s13049-019-0672-9

**Published:** 2019-11-07

**Authors:** Karen Alstrup, Thea Palsgaard Møller, Lars Knudsen, Troels Martin Hansen, Jens Aage Kølsen Petersen, Leif Rognås, Charlotte Barfod

**Affiliations:** 1 Department of Research and Development, Pre-hospital Emergency Medical Services, Central Denmark Region, Olof Palmes Allé 34, 8200 Aarhus N, Denmark; 2 Department of Anaesthesiology, Aarhus University Hospital, Aarhus, Denmark; 3 The Danish Air Ambulance, Aarhus, Denmark; 4 Copenhagen Emergency Medical Services, University of Copenhagen, Aarhus, Denmark

**Keywords:** Helicopter EMS, Population, National, Diagnostics, Severity score, Critical care interventions

## Abstract

**Background:**

A national Helicopter Emergency Medical Service (HEMS) was introduced in Denmark in 2014 to ensure the availability of physician-led critical care for all patients regardless of location.

Appropriate dispatch of HEMS is known to be complex, and resource utilisation is a highly relevant topic. Population-based studies on patient characteristics are fundamental when evaluating and optimising a system. The aim of this study was to describe the patient population treated by the Danish HEMS in terms of demographics, pre-hospital diagnostics, severity of illness or injury, and the critical care interventions performed.

**Method:**

The study is a retrospective nationwide population-based study based on data gathered from the Danish HEMS database. We included primary missions resulting in a patient encounter registered between October 1^st^ 2014 and April 30^th^ 2018.

**Results:**

Of 13.391 dispatches registered in the study period we included 7133 (53%) primary missions with patient encounter: 4639 patients were air lifted to hospital, 174 patients were escorted to hospital by the HEMS physician in an ambulance, and in 2320 cases HEMS assisted the ground crew on scene but did not escort the patient to hospital. Patient age ranged from 0-99 years and 64% of the population were men. The median age was 60 years.

The main diagnostic groups were cardio-vascular emergencies (41%), trauma (23%) and neurological emergencies (16%). In 61% of the cases, the patient was critically ill/injured corresponding to a NACA (National Advisory Committee for Aeronautics) score between 4 and 7 (both included). In more than one third of the missions a critical care intervention was performed. Ultrasound examination and endo-tracheal intubation were the critical care interventions most frequently performed (21% and 20%, respectively).

**Conclusion:**

The national Danish HEMS primarily attends severely ill or injured patients and often perform critical care interventions. In addition, the Danish HEMS provides rapid transport to highly specialised treatment for patients in the more rural parts of the country.

Patients with cardio-vascular emergencies, trauma and neurological emergencies are among those patient groups most commonly seen.

We conclude that the overall dispatch profile appears appropriate but emphasise that continuous development and refinement is essential.

## Introduction

A national Helicopter Emergency Medical Service (HEMS) was introduced in Denmark in 2014 in addition to the ground EMS units ensuring that physician-led pre-hospital care is available for all patients regardless of their location [1]. HEMS offers fast initiation of advanced critical care on scene and during transportation as well as triage and rapid transport to highly specialised in-hospital care. It is, however, a limited and costly pre-hospital resource. Appropriate dispatch of HEMS units is therefore crucial in providing a safe and efficient service. HEMS dispatch is an area receiving an increasing amount of research attention, as cost-effectiveness and resource utilisation are highly discussed topics in health care.

Proper HEMS utilisation is known to be a complex and difficult task, and HEMS overtriage has been addressed in several studies [2–4] suggesting that it is a challenge affecting most emergency medical services.

Recently, our study group published a paper describing the design, available variables and data quality of the Danish HEMS database containing all HEMS dispatches [5]. Roughly, 60% of the dispatches resulted in a patient encounter, while the remaining dispatches were either cancelled in-flight or did not lead to take-off. Papers describing a complete national HEMS patient population are rare. The current paper is the first to describe the entire population of patients treated by the Danish HEMS in detail. Population-based studies provide information on patient characteristics and diagnoses and are essential when evaluating, planning and optimising a system. Therefore, knowledge of the HEMS mission and population profile is fundamental to improve not only dispatch and resource utilisation, but also patient safety and patient outcome.

The aim of the present study is to describe demographics, pre-hospital diagnoses, severity of illness/injury, and critical care interventions in all HEMS missions resulting in a patient encounter, thus providing benchmark figures and a basis for further research for a recently implemented pre-hospital resource.

## Methods

### Study design and population

This is a retrospective population-based study presenting prospectively collected data from the national Danish HEMS database. We included all primary missions resulting in a patient encounter registered between October 1^st^ 2014 and April 30^th^ 2018.

### Setting

Denmark is a mixed urban, semi-rural and rural relatively flat country of 45.000 km2 with a coastline of 8750 kilometres and more than 70 smaller islands not connected by road to the main land. It is divided into 5 health care regions with approximately 5,8 million inhabitants in total [6]. Each region has its own EMS agency including an Emergency Medical Dispatch Centre (EMDC) [7]. The regional EMS are responsible for dispatch, treatment, triage and transport of all patients from the emergency call is received at the dispatch centre to the patient has been handed over to the hospital staff or treatment has been completed on scene.

The Danish EMS system is based on 1) ambulances staffed by a combination of emergency medical technicians (EMTs) with either basic (EMT-B), intermediate (EMT-I) or paramedic (EMT-P) level of training, and 2) Rapid Response Vehicles (RRV) staffed by either single responder paramedics or pre-hospital critical care teams including a consultant anaesthesiologist and a paramedic, depending on regional differences.

The Danish HEMS system is 100% governmentally founded and free of charge at the point of use. In 2017 the annual costs were estimated at around 13.4 mill. EURO, which was the amount reported by the Danish HEMS organisation to the international Organisation for Economic Co-operation and Development (OECD). These costs include the running of the helicopters (operational and service costs) and the salary of the crews. Administrative costs, training and educational costs as well as running costs for drugs and equipment are excluded.

The service covers the entire country 24 hours a day 7 days a week by helicopters staffed by a consultant anaesthesiologist experienced in pre-hospital critical care, a pilot and a specially trained paramedic. During the study period the service operated three identical aircrafts (EC 135 P3). They are equipped and certified to operate under both visual and instrumental flight rules (VFR and IFR) as well as instrument meteorological condition (IMC) and night operations landing both on pre-surveyed landing sites and ad-hoc sites both day and night. For this we use Garmin GTN750 GPS-navigation and Euronav 7 moving map as well as night vision goggles. As one of the few civilian HEMS services in the world we use Point-In-Space (PinS) navigation in order to be able to fly to hospitals, HEMS bases and some of our pre-surveyed landing sites under IMC conditions. Ours is a single pilot operation but the HEMS paramedics are technical crew members trained to the level of non-flying pilots.

During the study period, the three aircrafts operated from three different bases (Ringsted, Billund and Skive).

All Danish EMS units are dispatched by the five EMDCs, in which healthcare professionals (specially trained nurses, ambulance technicians and paramedics) handle medical emergency calls from the public through the European emergency phone number 112. This includes assessment of the urgency and activation of the appropriate EMS response. Assessment of the appropriate response is based on a systematic interview with the caller supported by a criteria-based dispatch protocol [8, 9]. The Danish EMDCs and the EMS system has been described in detail elsewhere [8, 10, 11].

### The Danish HEMS dispatch criteria

HEMS dispatch is based on 1) immediate dispatch based on the 112 call, 2) crew request from the scene, 3) interhospital transfers, and 4) non-critical missions to smaller islands not connected by road to the mainland.

Immediate dispatches, crew requests as well as island missions are considered primary missions, whereas interhospital transfers define secondary missions. The interhospital transfers constitute 5-6% of the workload. Most frequently, patients are transferred between two intensive care units and in the vast majority of cases the receiving department is a highly specialised centre (e.g. stroke centre, centre for thoracic surgery, trauma centre).

Dispatch criteria are summarised in Table 1. In areas where the distance to one of the four university hospitals with specialised facilities (e.g. level 1 trauma centres, invasive cardiology, and stroke centre), is short, HEMS are infrequently dispatched.

**Table 1:** Dispatch criteria of the Danish HEMS

*1) Immediate dispatch based on the 112 call*	
Major trauma	
unconscious patient	
seriously injured patient	
high energy trauma	
entrapment	
massive haemorrhage	
Mass casualty	
Drowning	
Diving accident	
Time-critical conditions *or* admittance to a specific hospital centre is needed	
acute myocardial infarction/chest pain	
cardiac arrest	
stroke	
children suspected to suffer a life-threatening condition and HEMS is nearest physician-staffed resource	
suspected meningitis and HEMS is nearest physician-staffed resource	
significant burns	
traumatic amputation	
*2) Crew request from the scene*	
Patients to thrombectomy/thrombolysis (stroke/acute myocardial infarction)	
Admittance to a specific hospital centre is needed	
Additional critical care physician on scene is needed	
*3) Interhospital transfers*	
*4) Missions to islands not connected by road to the mainland*	

### Data source and data cleaning

Data were extracted from the Danish HEMS database which holds information on all HEMS dispatches. The database contains different report forms according to the type of mission [5]. In addition, telephone enquiries not leading to a mission is registered. Table 2 provides an overview and the definition of each mission type.

**Table 2:** The definition of each type of mission

*Air lifted patients*	Missions where the HEMS physician escorted the patient in the helicopter to the hospital
*Ground escorted patients*	Missions where the HEMS physician escorted the patient in an ambulance to the hospital
*Assisted patients*	Missions where HEMS attended the patient and assisted the ground crew (ambulance and RRV) but did not escort the patient
*Aborted missions*	Missions cancelled in-flight before reaching the scene
*Rejected missions*	Missions where take-off from the base did not occur

Telephone enquiries, aborted and rejected missions, as well as secondary missions were excluded from the analysis.

The database is characterized by a very high degree of data completeness due to regular surveys, educational efforts and by adding a visual warning indication system aiming to reduce the number of incomplete report forms [5]. Missing data for the main variables used in the study were: civil registry numbers 6,4% (a large proportion of these are patients not living in Denmark and therefore they do not have a civil registry number), patients missing a NACA score 0,1%, patients missing a diagnosis 0,2%, and missing values for the interventions 0,8%-4,2%. These missing data were not substituted.

In a recent study, we found possible misclassifications for 298 patients (e.g. a patient who has been air lifted by HEMS to hospital may also have a registration as e.g. being transported by ambulance or pronounced dead on scene, and patients assisted may have been registered as being air lifted by HEMS to hospital etc.). The first author manually surveyed these possible misclassifications by reading individual notes and free text evaluations entered by the HEMS physicians. Obvious misclassifications due to inconsistencies or errors in the report forms were corrected (n=268). One mission performed by a military search and rescue helicopter was not included.

### Descriptive variables

The pre-hospital diagnoses were divided into the following diagnostic groups based on the International Classification of Diseases, 10^th^ edition (ICD-10): cardio-vascular emergencies, neurological emergencies, respiratory emergencies, trauma, burns, poisoning, obstetrics, abdominal emergencies, and other medical conditions.

The severity of the patient´s illness/injury was evaluated based on the National Advisory Committee for Aeronautics (NACA) score. This score was modified in 1980 for use in severity scoring in pre-hospital settings and is currently used in the Danish HEMS. It has been found to correlate well with morbidity and mortality [12, 13]. The score ranges from 0-7 (Appendix 1). We divided the NACA score into two categories: NACA 0-3 and NACA 4-7. We considered a NACA score of 4, 5, 6 or 7 to represent a patient in a critical condition corresponding to a severe or critical illness or injury.

Critical care interventions reported in this study were endo-tracheal intubation (ETI), pre-hospital use of blood products, intraosseous cannulation (IO), automated chest compression device (ACCD), ultrasound examination (US) and pleural drainage (PD) which includes chest tube placement and thoracostomy.

Regarding ETI, all airway management is carried out as a rapid sequence induction except when intubating patients under ongoing cardio-pulmonary resuscitation as per standard operating procedure.

### Statistical analysis

Data obtained from the database were integrated into an Excel spread sheet, and further processed and analysed using Stata (Stata Statistical Software version 15.1, StataCorp, College Station, Texas, USA).

Results were reported as numbers, proportions and medians, including ranges or 95% confidence intervals, where relevant.

### Ethics

The study was approved by the Danish Data Protection Agency (No. 1-16-02-40-17) and by the Danish National Board of Health (No. 3-3013-2049/1).

According to the Act on Research Ethics Review of Health Research Projects, register-based studies do not require an approval from the research ethics committee system (No. 1-10-72-4-17).

## Results

**Fig. 1:**
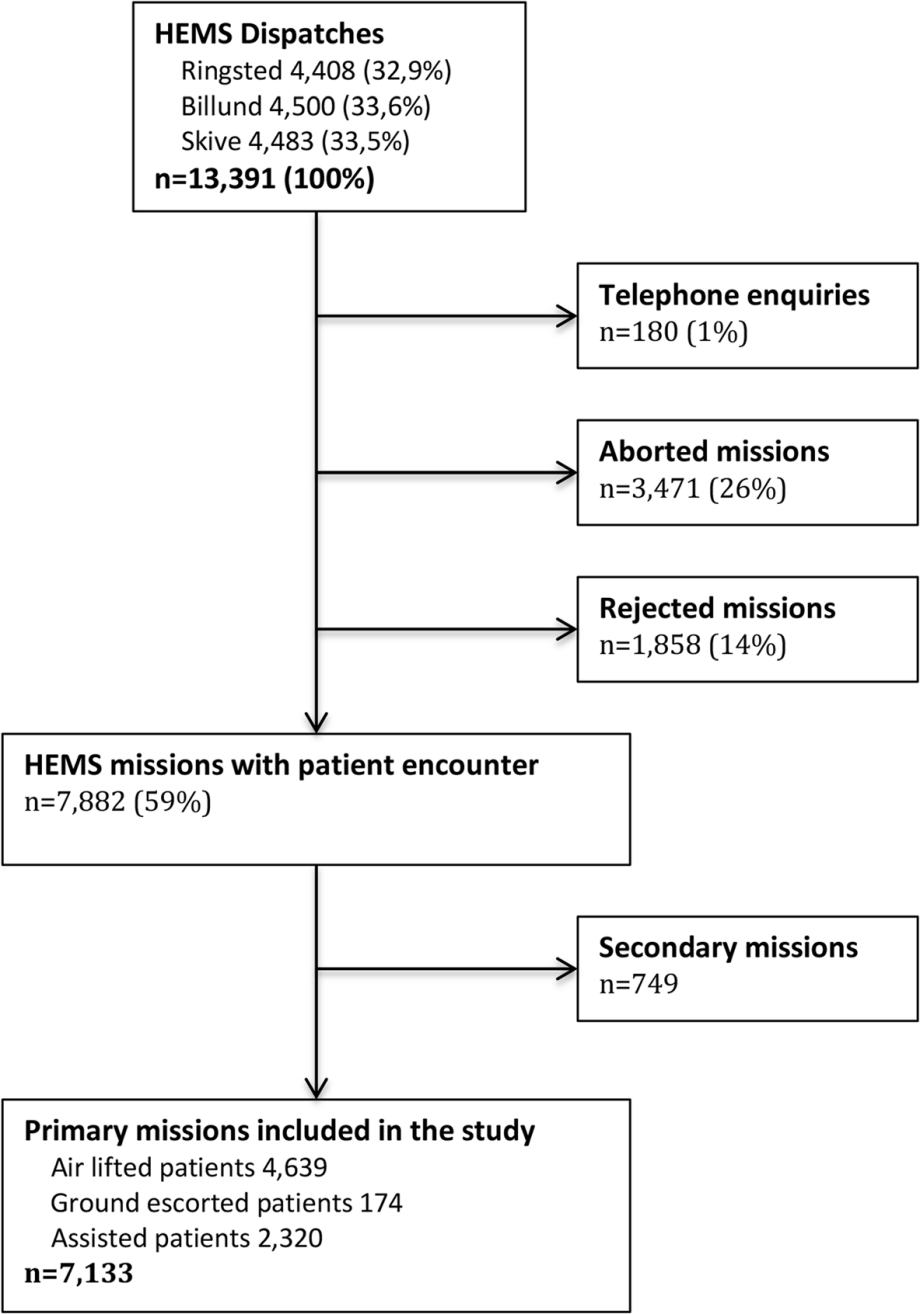
Flowchart showing all HEMS missions and the inclusion of patients

A total of 13.391 dispatches were registered in the database during the study period. The inclusion of patients is illustrated in Fig. 1. There were no differences in the utilisation of the three helicopters each accounting for one third of the dispatches. In 180 cases (1%), a telephone enquiry not leading to a mission was registered. Missions were aborted in 3471 cases (26%), and in another 1858 cases (14%) the mission was rejected. About half of those were due to adverse weather conditions. Thus, 7882 HEMS missions (59%) resulted in a patient encounter. 749 secondary missions were excluded leaving 7133 primary missions for further analyses. These missions were 4639 air lifted patients, 174 ground escorted patients and 2320 assisted patients.

The annual activity increased during the study period. In 2015, each helicopter was dispatched 2.8 times per day compared with 3.8 in 2017 and 4.3 in 2018 (from January 1^st^ until April 30^th^ 2018). Missions to an island not connected by road to the mainland represented 14% (n=976) of the cases.

**Fig. 2:**
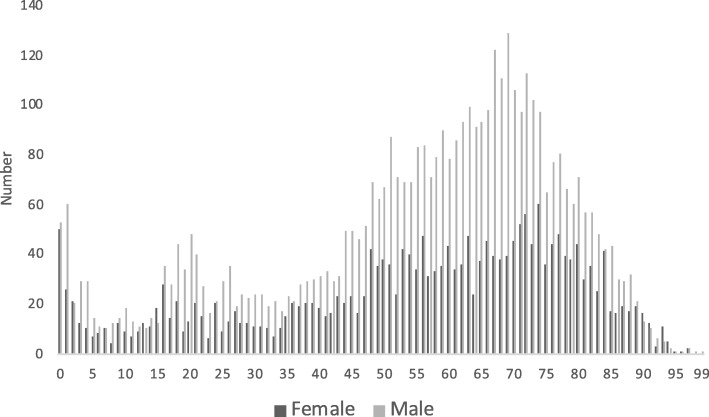
Age and gender distribution of the Danish HEMS patient population

Patient age ranged from 0-99 years. Fig. 2 shows the overall patient age and gender distribution. 64% of the population were men and their median age was 60 years (IQR: 44-71). Median age among women was 59 years (IQR: 40-73). We observed three peaks: children (≈0-2 years), young adults (≈16-26 years) and elderly (≈50-82 years). The distribution of patient gender and age according to the type of HEMS mission is presented in Table 3.

**Table 3:** Demographics and pre-hospital characteristics for 7,133 patients treated by the Danish Helicopter Emergency Medical Services (2014-2018)^a^

	Air lifted	Ground escorted	Assisted	**Total**
	patients	patients	patients	
	*n* = 4,639 (%)	*n* = 174 (%)	*n* = 2,320 (%)	***n*** = **7,133 (%)**
Gender				
Male (%)	3,051 (66%)	114 (66%)	1,381 (60%)	**4,546 (64%)**
Female (%)	1,468 (32%)	57 (33%)	818 (35%)	**2,343 (33%)**
Age		56,5 (32-72)		
median age, years (IQR)	61 (47-72)		56 (29,5-71))	**60 (42-72)**
<1 month (%)	16 (0,3%)	1 (0,6%)	14 (0,6%)	**31 (0,4%)**
1 month-1 year (%)	80 (2%)	8 (5%)	80 (3%)	**168 (2%)**
2-17 years (%)	270 (6%)	21 (12%)	200 (9%)	**491 (7%)**
18-66 years (%)	2,468 (53%)	82 (47%)	1,174 (51%)	**3,724 (52%)**
67+ years (%)	1,765 (38%)	60 (34%)	799 (34%)	**2,624 (37%)**
Pre-hospital diagnostic groups				
Cardio-vascular (%)	2,004 (43%)	54 (31%)	874 (38%)	**2,932 (41%)**
Acute myocardial infarction	1,030 (51%)^b^	2 (1%)^b^	8 (1%)^b^	1,040 (35%)^b^
Cardiac arrest	588 (29%)^b^	42 (24%)^b^	586 (67%)^b^	1,216 (41%)^b^
Neurology (%)	851 (18%)	29 (17%)	258 (11%)	**1,138 (16%)**
Respiratory (%)	121 (3%)	19 (11%)	63 (3%)	**203 (3%)**
Trauma (%)	999 (22%)	42 (24%)	592 (26%)	**1,633 (23%)**
Burns (%)	121 (3%)	4 (2%)	28 (1%)	**153 (2%)**
Poisoning (%)	9 (0,2%)	0	13 (0,6%)	**22 (0,3%)**
Obstetric (%)	6 (0,1%)	0	1 (0,04%)	**7 (0,1%)**
Abdominal (%)	113 (2%)	2 (1%)	42 (2%)	**157 (2%)**
Other medical condition (%)	415 (9%)	24 (14%)	449 (19%)	**888 (12%)**
Severity according to NACA score				
NACA 0 (%)	2 (0,04%)	0	14 (0,6%)	**16 (0,2%)**
NACA 1 (%)	7 (0,2%)	1 (0,6%)	97 (4%)	**105 (2%)**
NACA 2 (%)	143 (3%)	3 (2%)	363 (16%)	**509 (7%)**
NACA 3 (%)	1,205 (26%)	29 (17%)	906 (39%)	**2,140 (30%)**
NACA 4 (%)	1,983 (43%)	68 (39%)	239 (10%)	**2,290 (32%)**
NACA 5 (%)	678 (15%)	30 (17%)	26 (1%)	**734 (10%)**
NACA 6 (%)	596 (13%)	41 (24%)	44 (2%)	**681 (10%)**
NACA 7 (%)	22 (0,5%)	2 (1%)	627 (27%)	**651 (9%)**
NACA score - non-critical vs. critical emergency				
NACA 0-3 (%)	1,357 (29%)	33 (19%)	1,380 (59%)	**2,770 (39%)**
NACA 4-7 (%)	3,279 (71%)	141 (81%)	936 (40%)	**4,356 (61%)**
Intervention				
Intubation (%)	1,082 (23%)	48 (28%)	329 (14%)	**1,459 (21%)**
by HEMS	626	41	213	880
by other	456	7	116	579
Blood administration (%)	141 (3%)	7 (4%)	47 (2%)	**195 (3%)**
Intraosseous access (%)	255 (6%)	15 (9%)	192 (8%)	**462 (7%)**
ACCD (%)	297 (6%)	21 (12%)	210 (9%)	**528 (7%)**
Ultrasound examination (%)	932 (20%)	51 (29%)	558 (24%)	**1,541 (22%)**
Chest tube/thoracostomi (%)	28 (0,6%)	2 (1%)	10 (0,4%)	**40 (0,6%)**
At least one intervention performed (%)	1,636 (35%)	85 (49%)	707 (30%)	**2,428 (34%)**
Mission outcome for ground assisted patients (n=2320)				
Completed on scene (%)			144 (6%)	
Admitted to hospital by ambulance (%)			1,171 (51%)	
Admitted to hospital by paramedic/nurse (%)			155 (7%)	
Escorted by rapid response vehicle (%)			222 (10%)	
Patient dead on scene (%)			46 (2%)	
Patient inaccessible			1 (0,04%)	
Standby			2 (0,1%)	
Patient pronounced dead on scene (%)			576 (25%)	
No information			3 (0,1%)	
Mission to an Island not connected by road to the mainland				
Total (%)	867 (19%)	3 (1,7%)	106 (5%)	**976 (14%)**

^a^The total number of measurements in each of the variables is not in agreement with the total number of patients in the three groups due to missing data. Missing data varied from 0,1-4,3% across the variables

^b^The per centages presented for AMI and cardiac arrest represent proportions in relation to the cardio-vascular diagnostic group

### Pre-hospital diagnoses

Table 3 shows that the most frequent diagnostic group were cardio-vascular emergencies followed by trauma and neurological emergencies in all patient groups. In total, these diagnostic groups constituted 80% of the diagnoses in the study population.

Among the air lifted patients 51% were diagnosed with an acute myocardial infarction (AMI) and 29% suffered a cardiac arrest. This profile differed from the assisted patient group in which the majority (67%) suffered a cardiac arrest and few had acute myocardial infarction (1%).

Among the ground escorted patients, respiratory emergencies represented 11% compared to a mere 3% in both the air lifted and the assisted group. There was no difference in the trauma profile across the three groups.

### Severity of illness/injury

In Table 3 the distribution of patients according to each NACA score and the two categories (0-3 and 4-7) are summarised. Of all primary missions, 61% of the patients were classified as being severely ill/injured (NACA 4-7).

Among air lifted and ground escorted patients NACA 4-7 represented the majority of cases (71% and 81%, respectively).

In contrast, among the assisted patients the majority (59%) were assigned NACA 0-3.

Overall, few patients were assigned NACA 0, 1 or 2. Among the assisted patients NACA 3 was used the most (39%), but also a large proportion of the air lifted (26%) and ground escorted (17%) patients were assigned NACA 3. In both the air lifted and ground escorted patient group NACA 4 was the most frequently used score. Few assisted patients were classified as NACA 5 and 6. In contrast, almost all patients assigned NACA 7 were assisted patients.

**Fig. 3:**
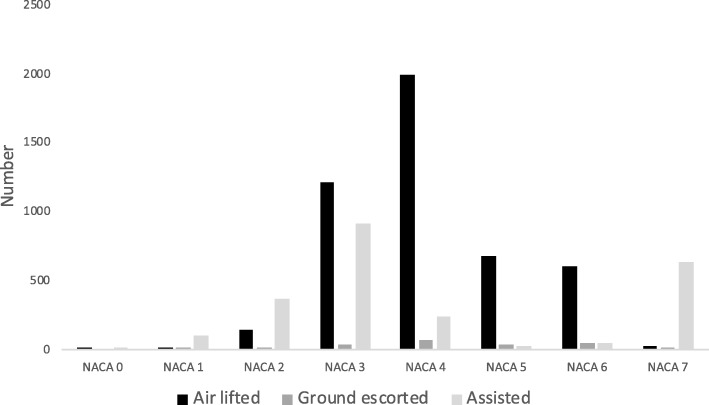
Number of patients with NACA score 0-7 according to the patient group

Fig. 3 shows the number of patients with NACA score 0-7 according to the patient group.

### Critical care interventions

The HEMS medical crews performed critical care interventions in 34% of all primary missions. The proportion of air lifted and assisted patients receiving at least one critical care intervention was almost similar (35% and 30%, respectively) and differed from the ground escorted patient group (49%).

Endo-tracheal intubation and ultrasound examination were the interventions most frequently performed (21% and 22% of all primary missions, respectively). Pleural drainage (chest tube placement/thoracostomy) were seldom performed (< 1% of all primary missions) and 3% of the patients were treated with blood products.

**Fig. 4:**
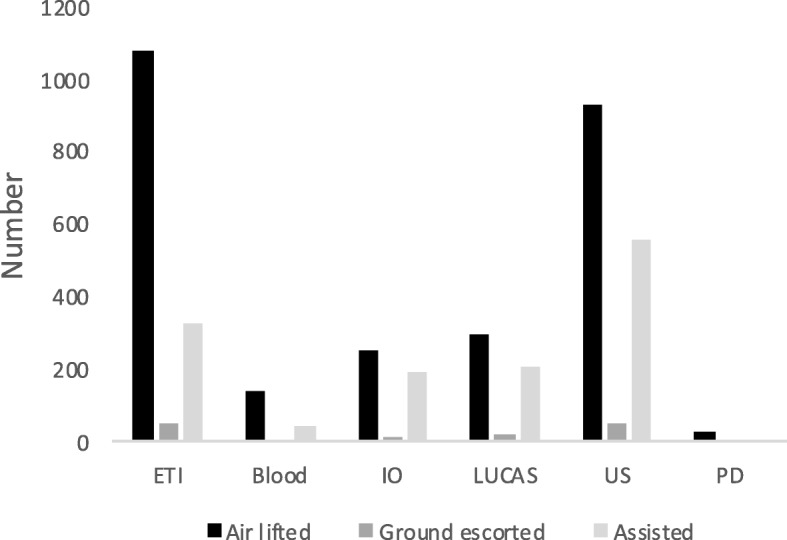
Distribution of critical care interventions performed. ETI; endo-tracheal intubation, IO; intraosseous cannulation, ACCD; automated chest compression device, US; ultrasound examination, PD; pleural drainage (chest tube/thoracostomy)

The distribution and number of interventions is presented in Fig. 4.

## Discussion

In this nationwide population-based study of 13.391 HEMS dispatches from October 1^st^ 2014 to April 30^th^ 2018, we included 7133 primary missions divided into 3 groups: air lifted, ground escorted and assisted patients.

We found an almost similar proportion of cardio-vascular emergencies among air lifted and assisted patients. For air lifted patients 80% of the cardio-vascular emergencies were represented by an AMI or a cardiac arrest. The high number of cardiac arrest patients in the assisted group likely represent patients in whom the resuscitation attempt was terminated on scene. The remaining one third of HEMS responses to cardio-vascular emergencies among the assisted patients, could in part reflect the difficult distinguishing between life-threatening and non-life-threatening cardio-vascular emergencies for the medical dispatcher.

Notably, the proportion of trauma patients in the three groups were similar. The fact that HEMS assisted but did not escort 592 trauma patients might indicate either an inappropriate dispatch of HEMS to minor traumatic cases or an appropriate dispatch to severe trauma patients where the resuscitation attempt was unsuccessful. This is a topic that deserves further exploration, but was beyond the scope of this study. Proper dispatch to severe trauma cases has proven a particular challenge [14]. Due to uncertainty on scene following a traumatic event, only limited information may be available for the dispatcher. In these situations, immediate dispatch based on the mechanism of injury, in contrast to dispatch based on a thorough systematically evaluation of the patient symptomatology, seems rational. This dispatch strategy has been found to reduce undertriage, but may contribute to overtriage [15, 16].

The decision made by the HEMS physician to escort a patient in an ambulance from the scene may be due to adverse weather conditions or lack of time gain by using the helicopter. Furthermore, in cases where the patient´s condition requires closer observation and easier access to the entire patient and/or interventions en route than what is possible in a noisy and compact aircraft ground escort may be preferred. A higher proportion of respiratory emergencies was observed among the ground escorted patients which could be due to impending airway compromise or respiratory failure.

HEMS attended severely ill/injured patients (NACA 4-7) in 61% of all missions. The NACA score distribution for air lifted and ground escorted patients were similar, and both differed substantially from the assisted patient group. The many patients in the assisted group (59%) assigned NACA 0-3 have a questionable need for highly specialised care or rapid transportation, and may indicate HEMS overtriage. The reported mission outcome for assisted patients (Table 3) could support this as 58% were admitted to hospital by non-physician-staffed EMS units. The critically ill/injured assisted patients assigned NACA 4, 5 and 6 (13%) may represent patients escorted to hospital by the physician-staffed rapid response vehicles. This topic requires further analysis.

Notably, also a large proportion of air lifted patients were assigned NACA 0-3. These missions may, in part, be explained by dispatches to islands where logistics rather than the clinical condition of the patient has priority.

More than one third of HEMS missions resulted in at least one critical care intervention, particularly endo-tracheal intubation and ultrasound examination. This proportion of interventions has also been reported in other HEMS studies, although the definitions of a critical care intervention varied [17, 18]. Although the physicians did not specify what kind of ultrasound examination they performed (eFAST, FATE), the examination was included in this study as a critical care intervention as it may distinguish between, or rule out, specific emergency conditions such as ruptured aortic aneurysm, acute cardiac failure and pulmonary diseases, impacting both on scene treatment and decision-making in triage and escort [19, 20].

In the literature, critical care interventions and the NACA score has been suggested as proxy markers for HEMS mission relevance [21]. In a recently published Scottish study, the authors found an overall 42% of HEMS missions appropriately tasked based on performed critical care interventions [22]. However, HEMS may be correctly dispatched at a lower threshold to patients not severely ill/injured (e.g. island evacuations in our setting), or to patients who do not need pre-hospital critical care interventions (e.g. patients suffering from stroke or acute myocardial infarction) but need rapid and safe transport to highly specialised and centralised hospital treatment. Thus, the number and type of critical care interventions and the NACA score are all valuable elements in the description of the HEMS population, but may not be the only relevant parameters when evaluating HEMS dispatch accuracy and mission relevance. A subset of patients may benefit from rapid transfer to specialised care, while other patient groups may benefit from critical care interventions provided on scene and during transport. Some patients need both.

The increase in HEMS caseload, especially during the first years of service, likely reflect organisational inexperience, different dispatch procedures and adjustment of dispatch protocols. According to the dispatch protocol, HEMS should be restricted to patients suspected of being critical ill/injured, and further, should assist in interhospital transfers as well as patient evacuations from islands with limited EMS transport capabilities.

Based on the HEMS activity, the diagnostic profile and NACA score distribution among the patients, the overall dispatch profile and a cancellation rate of 26% indicate a well implemented organisation including a well-trained dispatcher staff with a proper adherence to dispatch guidelines.

### Perspectives and future research

This paper is a rare evaluation of a complete national HEMS patient population; most other studies describe a single or a regional HEMS. The presented benchmark figures may be important in order to evaluate whether the current use of HEMS meets the overall political and operational goal. It is also a key tool when planning and optimising the service as it provides a valuable base-line for decision-makers in the setting of new priorities and visions bearing in mind that patient characteristics might change over time influencing these decisions (e.g. population ageing and diagnostic profile).

Our results may indicate an overuse of HEMS for seemingly minor traumatic incidents and non-life-threatening cardio-vascular emergencies, and a deeper insight into the triage process of HEMS to these cases may be of value.

Knowledge of the severity of the trauma patients attended to by the Danish HEMS is limited due to the fact that injury scoring (Injury Severity Scores or Abbreviated Injury Scores [23]) of trauma patients is not systematically reported in our system. This topic deserves a thorough evaluation.

Also, a further investigation of the many HEMS dispatches to patients with an apparent severity score may add valuable information to the assessment of overall dispatch accuracy.

The substantial proportion of HEMS missions to patients on islands not connected to the mainland was somewhat expected according to the island dispatch criteria. However, more insight into the island patient population in terms of demographic and socio-economic differences and outcomes after HEMS transportation would add knowledge to the planning and prioritising of resources.

Lastly, this study focuses on pre-hospital characteristics of the HEMS patient population, and an evaluation of in-hospital diagnostics, morbidity and mortality would provide a more comprehensive description of the population.

### Strengths and limitations

A major strength of the study is the nationwide population-based design including all three HEMS units and five EMDCs providing a complete picture of the Danish HEMS patient population. Moreover, as the Danish healthcare system is based on free and tax-supported services, a genuine population-based study is ensured.

The data quality is considered high [5]. The HEMS database is characterized by a high degree of data completeness and uniformity in the registration procedure, and therefore acts as a valuable tool for research in HEMS related critical care and dispatch.

However, the study has several limitations including its observational design. Patient assessment and treatment in a pre-hospital setting is complex and influenced by multiple factors. Inter-rater variability is therefore inevitable, and this may influence on a clear and stringent data registration, and thus, the interpretation of results.

Furthermore, missing data, although within a limited range for the reported variables, can also bias the results if systematically skewed.

As the study is carried out on highly specialised pre-hospital units mostly serving rural parts of Denmark and the islands, the generalisability may be restricted to other pre-hospital critical care services with similar staffing, caseload and case mix.

## Conclusion

The national Danish HEMS primarily attends severely ill or injured patients and often perform critical care interventions. In addition, the Danish HEMS provides rapid transport to highly specialised treatment for patients in the more rural parts of the country.

Patients with cardio-vascular emergencies, trauma and neurological emergencies are among those patient groups most commonly seen.

We conclude that the overall dispatch profile appears appropriate but emphasise that continuous development and refinement is essential.

## Supplementary information

**Additional file 1:** The National Advisory Committee for Aeronautics scoring system.

## Abbreviations

HEMS: Helicopter Emergency Medical Services
EMDC: Emergency Medical Dispatch Centre
EMT: Emergency Medical Technician
RRV: Rapid Response Vehicle
NACA: National Advisory Committee for Aeronautics score
ETI: Endo-tracheal intubation
IO: Intraosseous cannulation
ACCD: Automated chest compression device
US: Ultrasound examination
PD: Pleural drainage (chest tube placement or thoracostomy)
