# Supplementary material for: Characteristics of patients treated by the Danish Helicopter Emergency Medical Service from 2014-2018: a nationwide population-based study
Source: Scand J Trauma Resusc Emerg Med. 2019 Nov 7;27:102. doi: 10.1186/s13049-019-0672-9 (PMC6836366; doi:10.1186/s13049-019-0672-9)
Supplement: Supplementary file 1 — Additional file 1: The National Advisory Committee for Aeronautics scoring system. [file 13049_2019_672_MOESM1_ESM.docx]

Appendix 1

| The NACA score system |  |
| --- | --- |
| No disease or injury | 0 |
| Minor disturbance - no medical intervention | 1 |
| Slight/moderate disturbance | 2 |
| Moderate/severe disturbance | 3 |
| Serious/potential life-threatening disturbance | 4 |
| Acute life-threatening disorder | 5 |
| Acute life-threatening disorder with manifest organ failure | 6 |
| Death | 7 |
